# The diagnostic value of fine-needle aspiration cytology in the early diagnosis of pulmonary cryptococcosis

**DOI:** 10.1590/S1678-9946202668033

**Published:** 2026-05-18

**Authors:** Dongxia Wang, Ning Wang, Jiejing Liu, Chengyan Zhao, Xue Xing

**Affiliations:** 1 The Second Affiliated Hospital of Dalian Medical University, Department of Clinical Laboratory, Dalian, Liaoning, China; 2 The Second Affiliated Hospital of Dalian Medical University, Department of Pathology, Dalian, Liaoning, China

**Keywords:** Pulmonary cryptococcosis, Fine-needle aspiration cytology, Early diagnosis, Diagnostic methods

## Abstract

Pulmonary cryptococcosis, an invasive fungal infection caused by *Cryptococcus* spp., is often misdiagnosed as tuberculosis or lung cancer due to overlapping clinical and radiological features, leading to treatment delays. In this descriptive study, we aim to characterize the diagnostic findings and clinical utility of fine-needle aspiration cytology (FNAC) in a series of patients with pulmonary cryptococcosis, within the context of other available diagnostic modalities. We retrospectively analyzed 10 patients with pulmonary cryptococcosis who underwent imaging-guided percutaneous lung aspiration. Wright-Giemsa-stained cytology smears were examined under oil immersion, enabling clear visualization of the characteristic morphological features of *Cryptococcus*. In this case series, FNAC provided a rapid cytological diagnosis within two hours in all 10 cases, consistent with the results obtained by metagenomic next-generation sequencing (mNGS) and serological testing. In contrast, conventional smear microscopy showed lower detection rates, and histopathology required longer processing times. The use of FNAC facilitated early diagnosis, enabling timely initiation of antifungal therapy and helping to avoid unnecessary surgical interventions. Our findings suggest that cytomorphological evaluation by FNAC is a rapid and valuable diagnostic tool in the early clinical management of pulmonary cryptococcosis, effectively complementing existing diagnostic methods.

## INTRODUCTION

Pulmonary cryptococcosis (PC) is a lung fungal infection caused by *Cryptococcus*, and is increasingly recognized in immunocompetent individuals, with 60% of cases occurring in human immunodeficiency virus (HIV)-negative patients^1,2^. Symptoms are often mild or absent, and lesions may be detected incidentally. Its radiographic features can mimic those of tuberculosis and lung cancer, leading to misdiagnosis and unnecessary surgeries^3^. Early and accurate diagnosis of PC is crucial for establishing appropriate antifungal treatment and avoiding invasive procedures.

Fine-needle aspiration cytology (FNAC) is a minimally invasive procedure commonly used to sample pulmonary lesions, especially to distinguish benign conditions from malignancies. While FNAC is well-established for diagnosing lung cancer, its application in infectious diseases such as cryptococcosis is less common. However, cytological smears can readily demonstrate microorganisms, and an experienced cytopathologist can often identify fungal organisms based on their morphology^4^. Case reports and small series have described the cytological diagnosis of cryptococcal infection at various sites (e.g., lymph nodes, thyroid, adrenal gland, and lung)^5,6^. Despite this, the role of cytology in the early diagnosis of PC has not been systematically evaluated, and clinicians may not routinely rely on it for definitive identification of fungal pathogens.

In our institution, aspiration cytology has served as a key tool for the early diagnosis of PC in several cases, facilitating timely clinical intervention. This descriptive study aims to report a series of 10 cases of PC infection, focusing on illustrating the practical application and cytomorphological findings of FNAC in the diagnostic workup. We describe the cytomorphological characteristics of *Cryptococcus* on Wright-Giemsa-stained smears and present diagnostic outcomes of FNAC alongside those obtained by histopathology, serological testing, metagenomic next-generation sequencing (mNGS), and conventional smear microscopy^7-11^. The aim is to characterize the role of FNAC within the diagnostic landscape, highlighting its potential as a rapid and practical method to support early diagnosis and improve PC management.

### Ethics

This study protocol was reviewed and approved by the Ethics Committee of The Second Affiliated Hospital of Dalian Medical University, approval Nº KY2025-392-01. The authors obtained informed consent from all patients, including consent for publication.

## MATERIALS AND METHODS

This study is a retrospective case series of 10 patients diagnosed with PC at our hospital between 2020 and 2024. All patients underwent non-contrast and contrast-enhanced chest computed tomography (CT) scans during hospitalization. CT-guided percutaneous lung puncture was performed by radiologists under sterile conditions using an 18-gauge needle to precisely target lesions, obtaining tissue cores (≥1 cm in length) and smear samples.

For microbial culture, samples were inoculated onto Sabouraud Dextrose Agar (SDA) and incubated at 28 °C. Typical yeast colonies generally appeared within two to seven days, and final identification was based on morphological assessment.

For cytological and histopathological analysis, smears were immediately prepared on glass slides for Wright–Giemsa staining. Tissue cores were fixed in 10% neutral buffered formalin, embedded in paraffin, and sectioned for staining with hematoxylin and eosin (H&E), periodic acid–Schiff (PAS), Grocott’s methenamine silver (GMS) stains, and mucicarmine stains.

Cryptococcal antigen testing was performed on serum samples using a commercial latex agglutination kit, with a titer of ≥1:4 considered positive.

For mNGS, nucleic acids were extracted from the same tissue or bronchoalveolar lavage fluid (BALF) samples. After library preparation, sequencing was performed on an Illumina platform. The generated reads were analyzed by aligning to a pathogen database for species identification.

## RESULTS

The cohort consisted of five males and five females, with a median age of 50 years. None of the patients were HIV-positive or severely immunocompromised; however, three had diabetes, four had hypertension, one was on corticosteroids, and one had a history of malignancy. The most common symptom was cough, with additional symptoms including low-grade fever and chest discomfort in some patients, while two were asymptomatic. Symptom duration ranged from two weeks to three months. CT scans revealed nodules or masses in all patients, with seven exhibiting solitary nodules and three presenting multiple bilateral nodules with 0.8-5.0 cm. A halo sign was observed in four cases, and cavitation was present in two. These findings initially raised suspicion of lung cancer in six patients and tuberculosis in two. Most cases were suspected to represent either malignancy or infection, prompting further diagnostic interventions such as biopsy or aspiration (Table 1).

**Table 1:** Cytomorphological diagnosis and corresponding pathological, serological, NGS, and fungal smear results

Case	Sex	Age	Lesion location	Admission diagnosis	CT	Cytomorphological diagnosis	Pathological diagnosis	Cryptococcal capsular polysaccharide antigen	NGS	Fungal smear
1	Female	50	Right lower lobe	Suspected pulmonary lesion; hypertension (grade 3, very high risk)	Multiple nodular and strip-like shadows in the right lower lobe, suggestive of tuberculosis	Abundant necrotic tissue, multinucleated giant cells, and fungal-like structures; suggestive of fungal infection with atypical epithelial cell hyperplasia	Necrotic tissue with encapsulated fungi, favoring *Cryptococcus*; special staining: AB-PAS (+), GMS (+), Mucicarmine (+)	Positive	/	Sputum culture negative
2	Male	65	Right lower lobe	Suspected pulmonary lesion; arrhythmia, atrial fibrillation; hypertension (grade 2, moderate risk)	Multiple nodular lesions in the right lower lobe, possibly inflammatory or neoplastic	Presence of fungi, multinucleated giant cells, and inflammatory cells; indicative of fungal pneumonia	Granulomatous inflammation with *Cryptococcus*-like fungi; special staining: PAS (+), GMS (+), Mucicarmine (+)	Weakly positive	/	/
3	Female	59	Left lower lobe	Suspected pulmonary lesion; hypertension (grade 3, very high risk); rheumatoid arthritis; hypothyroidism	Bilateral patchy and nodular lesions, increased compared to previous imaging, suggestive of inflammation	Multinucleated giant cells with intracellular fungal-like structures; suggestive of fungal infection	*Cryptococcus*-like pathogens detected in bronchial mucosal tissue; special staining: PAS (+), GMS (+), Mucicarmine (+)	Weakly positive	Negative (CSF)	Negative in sputum, bronchoalveolar lavage fluid (BALF), and CSF smears
4	Female	50	Left lower lobe	Left pulmonary mass; type 2 diabetes	Few hazy subpleural arc shadows in both lungs. Irregular mass near left interlobar pleura. Multiple bilateral pulmonary nodules	Multinucleated giant cells and fungi observed; suggestive of *Cryptococcus* infection	Granulomatous lesion with refractile, oval fungal spores; special staining: PAS (+), GMS (+), Mucicarmine (+)	Positive	/	Negative (Sputum culture)
5	Female	66	Right lower lobe	Right pulmonary mass	Dense, patchy opacity in the right lower lobe; lymphadenopathy	Multinucleated giant cells and intracellular fungi observed; suggestive of *Cryptococcus* infection	Granulomatous lesion with alveolar epithelial proliferation; special staining: PAS (+), GMS (+), Mucicarmine (+)	Negative	*Cryptococcus* neoformans detected in lung biopsy	/
6	Male	47	Right upper lobe	Pulmonary infection; type 2 diabetes	Bilateral multiple nodular lesions, some showing cavitation	Multinucleated giant cells engulfing fungal structures; suggestive of *Cryptococcus* infection	Chronic inflammatory infiltration with fibrosis; special staining: PAS (-), GMS (+), Mucicarmine (+)	Positive	*Cryptococcus* neoformans detected in BALF	Negative (Sputum and lung aspiration smear)
7	Male	66	Left upper lobe	Suspected pulmonary lesion; chronic angle-closure glaucoma; senile cataract	Patchy and consolidative lesions in the left upper lobe, increased density over time	Multinucleated giant cells and fungi observed; suggestive of *Cryptococcus* infection	Granulomatous changes with *Cryptococcus*-like organisms; special staining: PAS (+), GMS (+), Mucicarmine (+)	Positive	/	Negative (Sputum and lung aspiration smear)
8	Male	30	Left upper lobe	Suspected pulmonary lesion; hypertension (grade 2, moderate risk); fatty liver; left kidney stone	Indeterminate left upper lobe lesion, malignancy not excluded	Granulomatous inflammation with intracellular fungal spores; suggestive of *Cryptococcus* infection	Fibrotic changes without clear fungal elements; special staining: PAS (-), GMS (+), Mucicarmine (+)	Negative	*Cryptococcus* detected in lung biopsy	/
9	Female	71	Left lower lobe	Postoperative pancreatic cancer with abdominal lymph node metastasis; type 2 diabetes	Multiple bilateral nodules, suspected metastatic tumors	Necrotic tissue with fungal spores, multinucleated giant cells; suggestive of *Cryptococcus* infection	Granulomatous inflammation with *Cryptococcus*-like organisms; special staining: PAS (+), GMS (+), Mucicarmine (+)	Positive	/	/
10	Male	50	Right upper lobe	Pneumonia; suspected necrotizing pneumonia; hypokalemia	Progressive right upper lobe consolidation, possible abscess formation	Cryptococcus-like budding organisms and multinucleated giant cells; suggestive of *Cryptococcus* infection	Fibrotic changes with possible fungal or microbial elements, special staining: PAS (+), GMS (+), Mucicarmine (+)	Negative	*Cryptococcus* gattii detected in BALF	Positive (Lung aspiration smear)

/ = Not performed.

**Figure 1:**
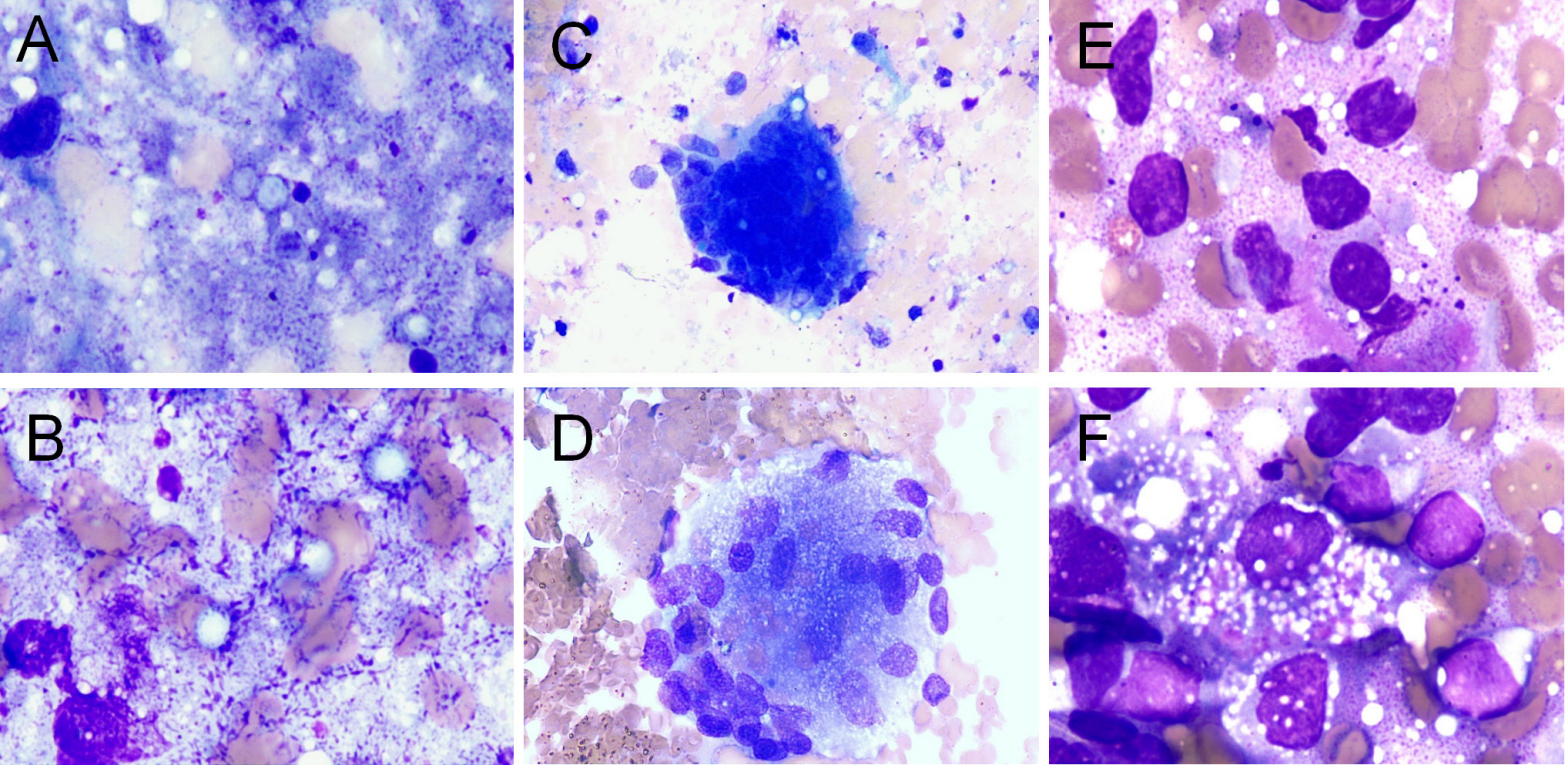
Inflammatory background in fine-needle aspiration (FNA) cytology smears of pulmonary cryptococcal infection: (A, B) necrotic tissue; (C, D) phagocytosis by multinucleated giant cells; (E, F) lymphocyte infiltration. All specimens were stained with Wright-Giemsa: A–D (×100), E, F (×1000).

**Figure 2:**
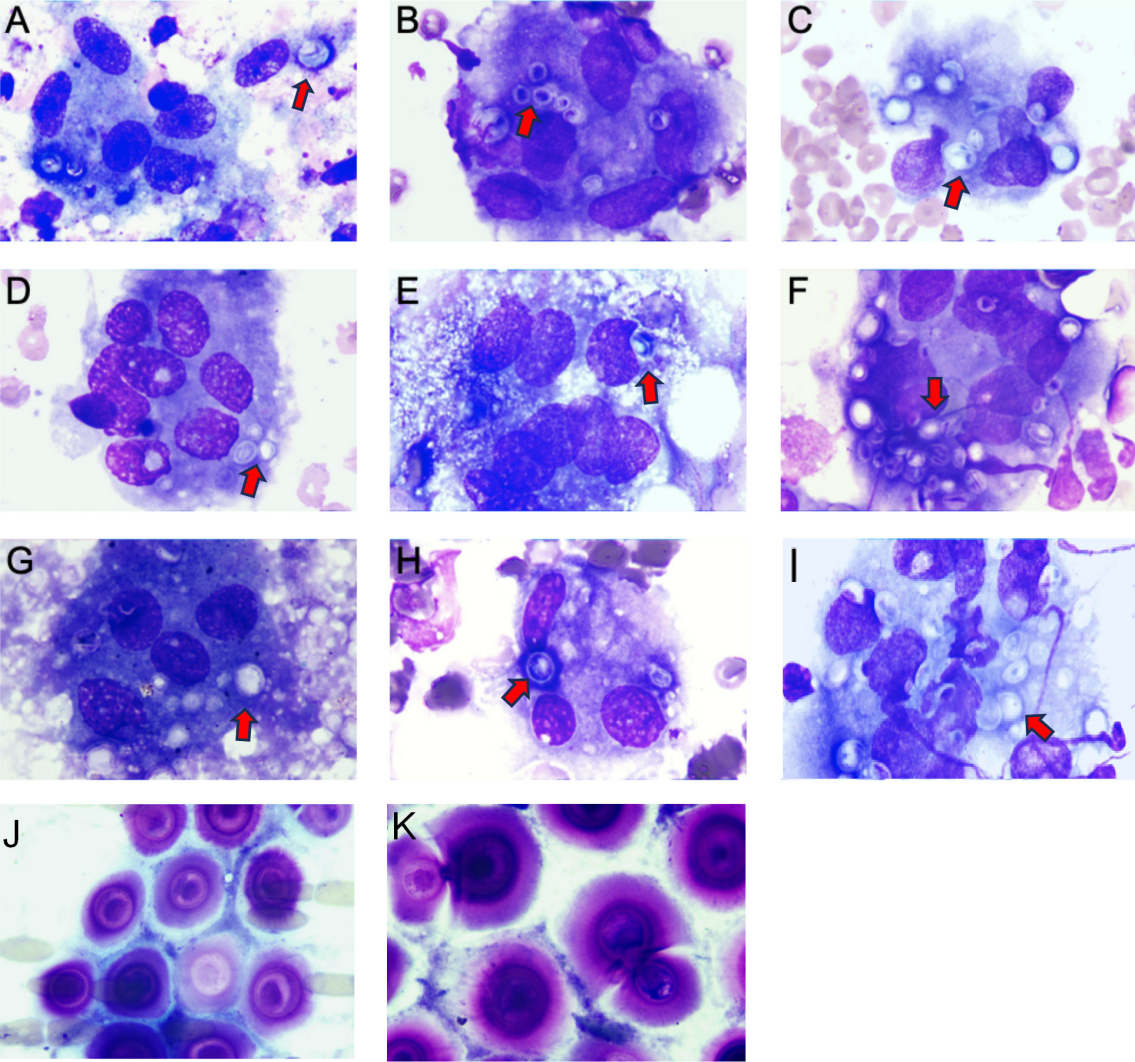
Morphological characteristics of pulmonary cryptococcal infection in puncture cytology: (A–I) representative morphological features of pulmonary cryptococcus in Patients 1–9 (indicated by the red arrow); (J, K) representative morphological features of *Cryptococcus gattii* in Patient 10. *Cryptococcus* organisms appeared in sheets, displaying large blue–purple structures surrounded by a prominent halo-like capsule. The inner cell wall was densely stained, occasionally appearing as a double ring. Narrow-based budding was observed, with pale or translucent daughter cells. Some organisms contained refractile cytoplasmic clear zones. All specimens were stained with Wright-Giemsa (×1000).

Cytopathological examination of rapid Wright-Giemsa-stained smears revealed fungal organisms with morphological features characteristic of *Cryptococcus* in all cases. The cytological smears exhibited a diverse cellular composition, often characterized by a mixed inflammatory background. Most cases presented with a moderate to abundant inflammatory infiltrate, consisting of neutrophils, macrophages, and lymphocytes (Figure 1). In three cases, multinucleated giant cells indicative of a granulomatous response were observed, primarily in immunocompetent hosts. *Cryptococcus* yeasts were observed either extracellularly or within macrophages, appearing as round to ovoid structures measuring 5-10 µm in diameter, with pale blue to purple cytoplasm on Wright-Giemsa staining. A distinct clear halo surrounding each yeast, representing the thick polysaccharide capsule, was also identified. Many yeasts displayed narrow-based budding, a characteristic morphological feature of *Cryptococcus*. No hyphal forms were detected in any of the cases, and the cytomorphology of *Cryptococcus* was illustrated in first nine cases (Figures 2A to 2I). However, Case 10 showed distinct morphological features. The background exhibited extensive necrosis with dense clusters of budding yeast cells. The organisms significantly differed from those observed in the other cases, appearing larger and more prominent (Figures 2J to 2K). mNGS identified *Cryptococcus gattii*.

Serological testing for cryptococcal antigen (CrAg) indicated a positivity rate of 70%. Notably, all negative cases occurred in immunocompetent patients with small, localized lesions, suggesting the potential for false-negative results. Conversely, positive cases presented with larger lesions or mild symptoms, underscoring the limited sensitivity of the test in isolated PC.

**Figure 3:**
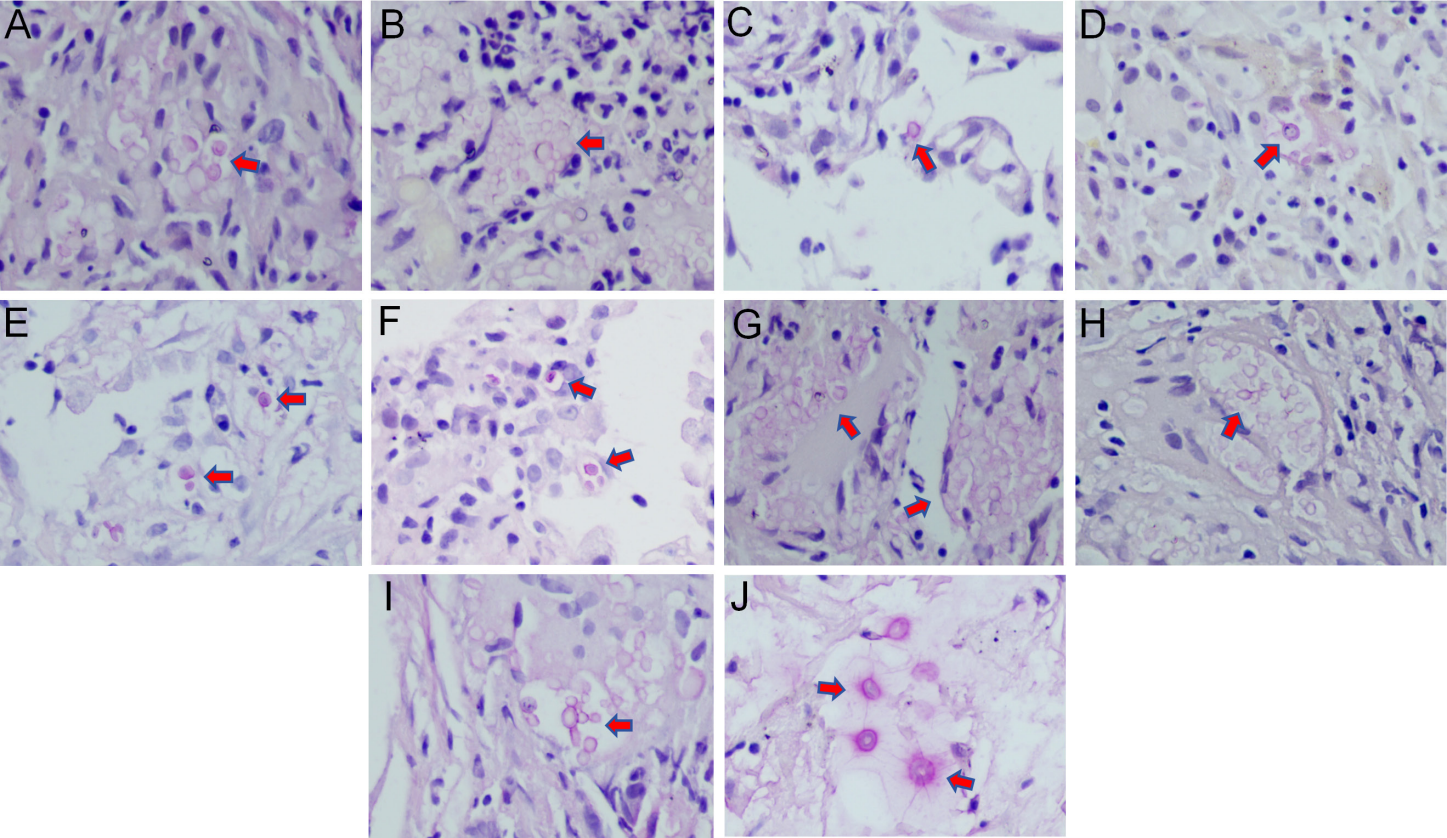
Mucicarmine staining of histopathological sections in pulmonary cryptococcosis: (A–J) representative mucicarmine-stained sections from Patients 1–10, showing characteristic staining of the cryptococcal capsule (indicated by the red arrow) (×400).

Histopathological examination, supported by comprehensive special staining, provided definitive confirmation in all cases. H&E staining revealed patterns of granulomatous inflammation or necrotic debris. GMS staining, performed in all cases, was positive in all 10 patients, conclusively demonstrating fungal cell walls. PAS staining was positive in eight of the 10 cases in which it was performed. The two initially PAS-negative cases (Cases 6 and 8) were GMS-positive, likely reflecting the focal nature of the infection and sampling depth; review of deeper tissue sections confirmed the presence of organisms. Furthermore, mucicarmine staining, employed to highlight the characteristic capsule, was positive in all cases, providing additional diagnostic specificity (Figure 3).

mNGS was used as a diagnostic tool in patients with suspected infection, successfully detecting *Cryptococcus neoformans* DNA in four of five tested patients, thereby supporting the cytological findings. Testing was not conducted in the remaining five patients due to various constraints. In one case, positive cytology was accompanied by a negative mNGS result, possibly due to cerebrospinal fluid sampling prior to fungal dissemination to the central nervous system, resulting in absent or undetectably low levels of fungal DNA^12^.

All patients commenced antifungal therapy after diagnosis. Nine patients were treated with oral fluconazole for PC, with a planned duration of six to 12 months. One patient received intravenous amphotericin B for two weeks before transitioning to fluconazole. Antifungal therapy was initiated immediately following cytological diagnosis when applicable, and no unnecessary surgical interventions were performed.

Follow-up evaluations revealed that eight out of nine patients exhibited improvement or resolution of lung lesions after treatment, with no reported relapses. These findings suggest that early diagnosis and timely therapeutic interventions can lead to favorable outcomes in patients with PC.

## DISCUSSION

PC poses a diagnostic dilemma due to its variable presentation and overlap with other pulmonary diseases^13,14^. The findings from this case series underscore the importance of using multiple diagnostic modalities and highlight the particular value of cytopathological examination of lung aspirates in achieving early diagnosis. In our series of 10 patients, FNAC proved to be a highly effective method, detecting *Cryptococcus* organisms in all cases, including those that might have been missed by conventional tests. To our knowledge, this is one of the few studies focusing on the role of aspiration cytology in the early and minimally invasive diagnosis of PC infection.

The high diagnostic yield of cytology in our series is notable. This may be attributed to several factors. First, *Cryptococcus* yeast cells are usually present in sufficient numbers within lesions, especially in necrotic or caseous material that can be aspirated. Second, their characteristic morphology—spherical yeasts with a capsule—enables identification on routine stains such as Wright-Giemsa or Papanicolaou, even before special stains are applied. In our experience, Wright-Giemsa staining was particularly useful for rapid on-site evaluation, as it clearly delineated the capsule as a halo and the yeast cell body as a basophilic round structure. This finding is consistent with prior reports in which Romanowsky-type stains (Diff-Quik, Giemsa) on cytology smears facilitated rapid recognition of cryptococci^6^. Third, the presence of an experienced cytopathologist who is aware of the possibility of fungal infection in any undiagnosed pulmonary nodule is crucial. In endemic areas or patient groups, a high suspicion index may lead to more careful examination of smears for organisms.

A critical consideration in the diagnosis of fungal infections via FNAC is the morphological differentiation among various yeast-like fungi. Pulmonary fungal infections may also be caused by *Blastomyces* species, *Histoplasma capsulatum*, and *Coccidioides* species. Therefore, a careful and comprehensive differential diagnosis is imperative.

*Cryptococcus* species present as round to oval yeast cells of variable size (2-20 µm), most notably characterized by a prominent, broad gelatinous capsule—a feature absent in the other three fungi. In contrast, *Blastomyces* species are typically large (8-15 µm, sometimes up to 30 µm) and uniform, with thick-walled cells that often exhibit a characteristic broad-based budding pattern. Conversely, *Histoplasma capsulatum* appears as small (2-4 µm), uniform yeast cells, typically observed within macrophages. It lacks a true capsule, and its small size and intracellular location are its most defining features. *Coccidioides* species, on the other hand, manifest in tissue as large spherules (20-100 µm in diameter) with thick walls containing numerous endospores (2-5 µm)—a morphology so distinctive that it is considered diagnostic when observed^15^.

Another key consideration is differentiating between *C. neoformans* and *C. gattii*, the two primary species causing cryptococcosis. Cytomorphology cannot reliably distinguish between these species, and definitive identification typically requires culture or molecular methods. In this study, one case of *C. gattii*, confirmed by NGS exhibited distinct microscopic features compared to several *C. neoformans* cases, particularly larger yeast cells, suggesting potential for preliminary differentiation. Based on epidemiological patterns, we presume most cases involved *C. neoformans*, although some *C. gattii* infections may have been present. Regardless, early recognition of the infection remains the priority^16^.

Beyond cytomorphological examination, histochemical staining remains a cornerstone for the definitive identification of fungal pathogens^17^. In two of our cases (Cases 6 and 8), initial PAS staining yielded negative results despite strong clinical and cytological suspicion. This finding underscores a fundamental diagnostic limitation: the sensitivity of any stain on a small biopsy specimen is inherently constrained by sampling. Fungal infections can be focal, and histological sections may not capture the organisms. In our series, deeper sections were subsequently obtained from the tissue blocks, and GMS staining—performed in all cases—reliably demonstrated fungal cell walls. For *Cryptococcus*, the prominent capsule is a key diagnostic feature. Mucicarmine staining, which specifically highlights this polysaccharide capsule, offers high diagnostic specificity and was positive in all cases in this series. Additionally, Fontana-Masson staining, which detects melanin in the cell wall, may be particularly useful in specific scenarios, such as when evaluating poorly encapsulated or intracellular yeast forms^18^.

Serological testing for cryptococcal antigen is a cornerstone in diagnosing cryptococcosis, particularly meningitis^19^. However, its role in isolated PC is less clear. The literature suggests that, in immunocompetent patients with localized disease, the serum CrAg test may be negative in a significant proportion of cases^9^. Our findings support this: 30% of our patients had negative antigen test results. Had we relied solely on noninvasive methods (antigen testing and imaging), nearly half of the diagnoses might have been missed or delayed. This underscores that a negative serum antigen result does not rule out PC and must be interpreted with caution. Conversely, all patients with positive antigen results in our series had confirmed cryptococcosis, confirming the high specificity of the test. Thus, a positive antigen result is highly informative, whereas a negative result should prompt further invasive diagnostic evaluation if clinical suspicion remains.

mNGS is an emerging technology that has begun to transform the diagnosis of infectious diseases^20^. In theory, mNGS can identify *Cryptococcus* DNA in respiratory samples even when traditional methods fail, and it has been reported to assist in diagnosing cryptococcosis in challenging cases^10^. In our practice, we found mNGS to be a useful adjunct; for instance, in one case, it confirmed the presence of the organism in the aspirate, supporting the cytological findings. However, the sensitivity of mNGS in real-world practice may not be 100%. Our cases indicate that factors such as sample type, DNA extraction efficiency, and fungal burden are critical. A negative mNGS result, as observed in some of our cases, cannot completely exclude infection. Moreover, mNGS typically requires specialized facilities and may take several days, whereas cytology can provide answers within hours. Therefore, while mNGS is a powerful tool, it should be considered complementary to, rather than a replacement for traditional diagnostic methods in the context of pulmonary fungal infections^11^.

Our results show that cytopathology can establish a diagnosis earlier than histopathology in many cases, with important clinical implications. Historically, the gold standard for diagnosing PC has been histopathological identification in lung tissue or culture confirmation. However, obtaining a surgical lung biopsy or resection is invasive and may not always be immediately feasible. By using FNAC, we were able to secure a diagnosis with minimal risk to the patient. In some cases, cytological evaluation helped avoid unnecessary thoracotomy or lobectomy that might have otherwise been performed under the presumption of lung cancer^21,22^. This aligns with recommendations that, in patients with isolated pulmonary nodules of unclear etiology (especially those with normal immune function), less invasive diagnostic procedures (such as needle biopsy or aspiration) should be pursued before surgical resection^3^. Early diagnosis via cytology enabled prompt initiation of antifungal therapy, which likely contributed to the favorable outcomes observed. For instance, one patient in our series started fluconazole a day after FNA, leading to symptom improvement within weeks. Had the diagnosis been delayed until histopathological or culture confirmation, treatment would have been delayed.

### Limitations

This study presents some limitations. The sample size is small, reflecting its single-institution, retrospective design and the relative infrequency of confirmed PC cases. Therefore, while our estimates of diagnostic sensitivity are illustrative, they should be interpreted with caution and may not be generalizable to all settings. Moreover, not all patients underwent every test (e.g., mNGS, smear microscopy, and culture), which may introduce bias in comparative analyses. However, we aimed to mitigate this limitation by focusing on overall trends and practical impact of each diagnostic approach. Finally, as a retrospective study, we relied on existing documentation and available samples; a prospective design may better capture differences in time to diagnosis and enable standardized testing across all patients.

## CONCLUSION

This study delineates the diagnostic utility of FNAC in PC. First, regarding diagnostic yield and cytomorphological features, FNAC demonstrated high efficacy by revealing the characteristic morphology of *Cryptococcus* in all cases using routine Wright-Giemsa staining. The yeast cells, often accompanied by a clear halo (representing the capsule), enable a rapid presumptive diagnosis. Second, in terms of clinical utility, this rapid cytological assessment is crucial. When correlated with clinical and radiological findings, it provides sufficient grounds to initiate timely antifungal therapy, which is essential for patient management. Furthermore, compared with other diagnostic modalities, FNAC offers a clear advantage in speed (often within hours) over histopathology and culture. However, it is most effective within an integrated diagnostic strategy. Its findings should be corroborated by more specific methods, such as histochemical stains (e.g., mucicarmine, GMS), cryptococcal antigen testing, or mNGS, for definitive confirmation and to guide targeted therapy. Therefore, we recommend that, in regions where PC is encountered, clinicians consider FNAC early in the evaluation of indeterminate pulmonary nodules, especially in immunocompetent individuals. A practical diagnostic approach may include FNAC for suspicious nodules; if suggestive cytology is found, treatment should be initiated promptly while confirmatory testing is pursued concurrently. This approach facilitates early intervention and may reduce patient morbidity associated with diagnostic delays or unnecessary invasive procedures.

## Data Availability

The data are available from the corresponding authors upon reasonable request.
